# Retrospective Analysis of Survival Improvement by Molecular Biomarker-Based Personalized Chemotherapy for Recurrent Ovarian Cancer

**DOI:** 10.1371/journal.pone.0086532

**Published:** 2014-02-05

**Authors:** Youngchul Kim, Saketh R. Guntupalli, Sun J. Lee, Kian Behbakht, Dan Theodorescu, Jae K. Lee, Jennifer R. Diamond

**Affiliations:** 1 Department of Public Health Sciences, University of Virginia, Charlottesville, Virginia, United States of America; 2 University of Colorado Cancer Center, University of Colorado Denver, Aurora, Colorado, United States of America; 3 Konkuk University School of Medicine, Seoul, Korea; H. Lee Moffitt Cancer Center & Research Institute, United States of America

## Abstract

Aggressive tumors such as epithelial ovarian cancer (EOC) are highly heterogeneous in their therapeutic response, making it difficult to improve overall response by using drugs in unselected patients. The goal of this study was to retrospectively, but independently, examine whether biomarker-based personalized chemotherapy selection could improve survival of EOC patients. Using *in vitro* drug sensitivity and patient clinical outcome data, we have developed *co-expression extrapolation* (COXEN) biomarker models for predicting patient response to three standard chemotherapy drugs used to treat advanced EOC: paclitaxel, cyclophosphamide, and topotecan, for which sufficient patient data were available for our modeling and independent validation. Four different cohorts of 783 EOC patients were used in our study, including two cohorts of 499 patients for independent validation. The COXEN predictors for the three drugs independently showed high prediction both for patient short-term therapeutic response and long-term survival for recurrent EOC. We then examined the potential clinical benefit of the simultaneous use of the three drug predictors for a large diverse EOC cohort in a prospective manner, finding that the median overall survival was 21 months longer for recurrent EOC patients who were treated with the predicted most effective chemotherapies. Survival improvement was greater for platinum-sensitive patients if they were treated with the predicted most beneficial drugs. Following the FDA guidelines for diagnostic prediction analysis, our study has retrospectively, yet independently, showed a potential for biomarker-based personalized chemotherapy selection to significantly improve survival of patients in the heterogeneous EOC population when using standard chemotherapies.

## Introduction

With an estimated 224,747 new cases and 140,163 deaths annually worldwide, epithelial ovarian cancer (EOC) is one of the most lethal gynecologic malignancies [1]. The basic treatment for advanced EOC has been surgical removal of disease and the subsequent use of platinum and taxane combination chemotherapy. While the vast majority of women with the disease achieve clinical remission with this treatment, over 80% suffer a recurrence. The results of the largest multinational trial for advanced ovarian cancer demonstrate that the uniform incorporation of additional cytotoxic agents into the platinum-taxane backbone does not significantly improve the progression-free interval in this patient population [2]. Advanced EOC is highly heterogeneous in therapeutic response, whereby only a small proportion of patients receive benefit from any given chemotherapeutic agent. For instance, if a hypothetical anticancer drug provided a one year improvement in survival for 20% of advanced EOC patients, the overall improvement in survival from its *unselected* use for the entire patient population would be a mere 2.4 months. On the other hand, if we can accurately select subsets of patients who can benefit from particular drugs, it may be possible to significantly improve overall survival from a selective use of the most effective drugs, while avoiding unnecessary drug toxicity for patients unlikely to derive meaningful clinical benefit.

Various single- and multi-gene biomarker developments have recently shown a high potential to predict cancer patient therapeutic response and survival. Gene expression biomarkers that were discovered from direct correlation with patient prognosis and clinical follow-up data significantly predicted the survival of breast cancer patients [3], [4]. The 93-gene signature developed with genomic expression profiling and clinical follow-up data from 60 ovarian cancer patients was highly predictive of a pathologic complete response to platinum-taxane chemotherapy [5]. Helleman et al. sought to predict resistance to platinum therapy by evaluating genomic data for 96 ovarian cancer patients, obtaining a nine-gene signature for platinum resistance [6]. Williams et al. developed gene expression models based on *in vitro* chemosensitivity information and microarray analysis of the NCI-60 cancer cell line panel, which were able to stratify responders from non-responders in diverse patient sets for ovarian and other cancers [7]. Ferriss et al. developed models predictive of single-drug response for carboplatin and paclitaxel in EOC by identifying common biomarkers between *in vitro* drug sensitivity and patient outcomes and further triaging the ones consistently expressed both in frozen and formalin-fixed paraffin embedded (FFPE) tissue samples [8]. The resulting predictors could successfully predict therapeutic responses to single-drug and combination chemotherapy, both from fresh-frozen and archived FFPE tumor samples from EOC patients. While these biomarker developments have shown high potential for molecular expression-based prediction of cancer patient chemotherapeutic response, they have not yet shown direct clinical benefits from the use of these molecular predictors.

Many clinical factors, such as tumor stage, age, surgical outcome, and other clinicopathological variables, have also been reported to be relevant to the success of therapeutics in EOC [9]. In this study, we have developed molecular biomarker models of single chemotherapeutic drugs by integrating *in vitro* drug sensitivity and patient clinical outcome data for consistently predicting therapeutic response and long-term survival of EOC patients treated with standard chemotherapy. Independently examining a possible personalized treatment use of these biomarker models on a large retrospective EOC patient cohort, we also show the potential of significant survival improvement for recurrent ovarian cancer.

## Methods

### Patient Data

*In vitro* drug activity and microarray data for the 60 NCI cancer cell lines (NCI-60) were previously described [10]. In brief, publicly available drug sensitivity data for 50% growth inhibition (GI_50_) for the NCI-60 were obtained from the NCI Developmental Therapeutics Program (http://dtp.nci.nih.gov). NCI-60 expression profiling data with HG-U133A GeneChip® arrays (Affymetrix, Santa Clara, CA) were also obtained from the National Cancer Institute (http://discover.nci.nih.gov). Microarray gene expression data for frozen tissue samples obtained at the time of primary cytoreductive surgery from two large human ovarian cancer cohorts were used for the development and independent evaluation of our drug-response predictors. Clinical follow-up information after surgery and chemotherapy were fully available for these cohorts. The first cohort of 185 EOC patients treated with adjuvant chemotherapy, **Bonome-185**, was originally obtained for identifying prognostic molecular signatures of survival [11]. Of 185 patients, 112 (67%) showed complete response (CR), 41 (25%) partial response (PR), 14 (8%) progression of disease (PD), and 18 had unrecorded responses to the primary chemotherapy (**Table 1**). The best response to chemotherapy was determined according to RECIST or WHO criteria at the completion of adjuvant chemotherapy [12], [13]. The second set of 448 epithelial ovarian cancer patients whose Affymetrix gene expression profiling and clinical follow-up data were available, **TCGA-448**, was obtained from The Cancer Genome Atlas (TCGA) consortium (http://tcga-data.nci.nih.gov) [14]. These EOC patients from >10 diverse clinical centers had received primary platinum-based chemotherapy after surgery. The primary chemotherapy responses of this cohort were comprised of 272 (60.71%) patients with CR, 54 with PR, 25 with stable disease (SD), and 36 with PD. However, a majority of the patients experienced recurrence or progression of disease and so were subsequently treated with additional chemotherapy drugs such as cyclophosphamide and topotecan. In particular, of 100 recurrent patients treated with topotecan, 47 patients were from the University of Washington (TCGA-UW) and the remaining 53 patients were from 11 other hospitals (TCGA-test). For the third cohort of 51 patients with stage III–IV EOC at the University of Virginia (**UVA-51**), gene expression data were obtained from archived FFPE tissue blocks, and both chemotherapy response and long-term survival information were available [15]. This cohort had 28 CR and 23 NR patients. The last cohort of 99 patients used in our study, **Wu-99**, was from a gene expression profiling study on a general EOC patient population prior to primary chemotherapy; we used this set to find initial biomarkers that were concordantly expressed between cancer cell lines and human patients [10]. More detailed clinical characteristics of these cohorts are summarized in **Table 1**. Bonome-185 and Wu-99 patient data were previously published elsewhere. The TCGA-443 patient data were obtained from the TCGA public domain. For the UVA-51 cohort, we obtained and used the archived patient samples and de-identified clinical data which were consented for general research purpose and approved by the Institutional Review Board (PRC# 455-07) at the University of Virginia; its full description has been published elsewhere [8].

**Table 1:** Epithelial ovarian cancer (EOC) patient cohorts for the development and validation of integrated predictors for patient response to standard chemotherapy drugs.

	Historical patient cohorts			
Characteristic	Bonome-185	TCGA-448	UVA-51	Wu-99
MedianAge(range)	63.6(26–85)	59 (27–87)	62 (44–84)	
Stage				
I	–	–	–	35 (35.4%)
II	–	24 (5.4%)	–	11 (11.1%)
III	144 (77.8%)	354 (79%)	46 (90.2%)	44 (44.4%)
IV	41 (22.2%)	68 (15.2%)	5 (9.8%)	9 (9.1%)
Others	–	2 (0.4%)	–	–
Histology				
Serous	166 (89.7%)	448 (100%)	42 (82.4%)	41 (41.4%)
Clear Cell	–	–	5 (9.8%)	8 (8.1%)
Others	19 (10.3%)	–	4 (7.8%)	50 (50.5%)
Surgical Outcome				
Optimal(<1 cm)	92 (49.7%)	291 (65%)	21 (41.2%)	
Sub-optimal(> = 1 cm)	93 (50.3%)	111 (24.8%)	28 (54.9%)	
Others(missing)	–	46 (10.3%)	2 (3.9%)	
Response toInitial Therapy				
CR	112 (60.5%)	272 (60.7%)	28 (54.9%)	
PR	41 (22.2%)	54 (12%)	22 (43.1%)	
SD	–	25 (5.6%)	–	
PD	14 (7.6%)	36 (12.1%)	1 (2%)	
Others	18 (9.7%)	61 (13.6%)	–	
Recurrence/Disease Free	–	332 (74.1%)	44 (58%)	
Deaths	145 (78.4%)	238 (53.1%)	31 (60.8%)	
Survival (month)				
Median PFS	>5.83	16.7	12.42	
Median OS	44.2	44.8	50.4	

### Statistical Analysis

**Figure 1:**
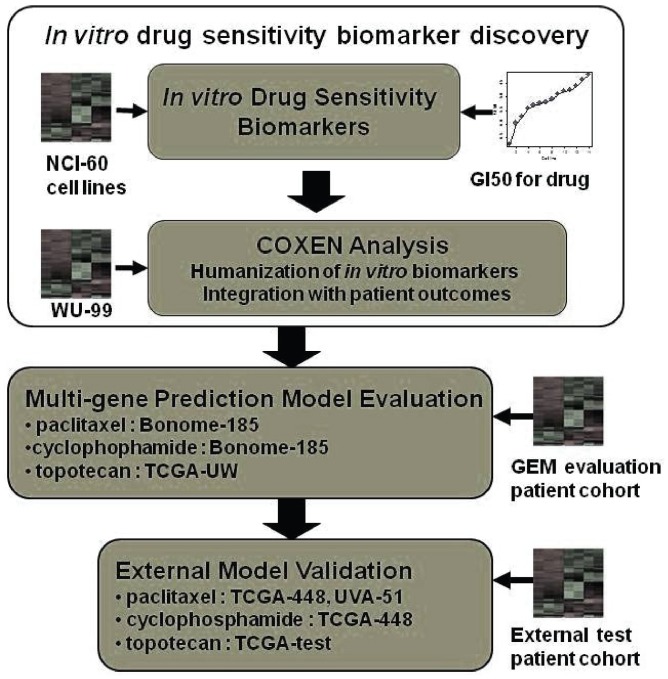
Integrated co-expression extrapolation (COXEN) gene expression model (predictor) development and validation procedures.

Multivariate models for predicting patient therapeutic responses to three chemotherapy drugs, paclitaxel, cyclophosphamide, and topotecan, were derived by integrating *in vitro* drug sensitivity data for the NCI-60 cell lines and clinical outcome information from EOC patients after standard chemotherapy. The schematic procedures for our model training and validation are summarized in **Figure 1**. First, initial gene expression biomarkers highly associated with *in vitro* drug sensitivity were identified from the NCI-60 microarray data by correlating each drug’s GI_50_ values for the NCI-60 with their genomic expression data for cyclophosphamide and topotecan treatment and by identifying differentially expressed biomarkers between sensitive and resistant cell lines of the NCI-60 to paclitaxel. These chemosensitivity biomarkers were then triaged based on the COXEN coefficient, which represents the degree of concordance of expression regulation between the NCI-60 cell lines and a general EOC patient population prior to standard chemotherapy [16]. In brief, derivation of the COXEN coefficient is based on a so-called “correlation of correlations,” which first calculates the expression correlations within each set for the identical set of genes and then evaluates gene-by-gene correlation between the correlation matrices of the two sets. This kind of second-order correlation has proven useful by us and others for investigating various gene networks to identify concordant data sets [17]–[19]. More detailed description of the COXEN algorithm can be found elsewhere [7], [10].

The above biomarkers were further screened with ovarian cancer patient data: the Bonome-185 set for paclitaxel and cyclophosphamide and the TCGA-UW set for topotecan. A subset of each drug’s biomarkers significantly associated with patient survival was identified by a Cox regression survival analysis. Therefore, these final biomarkers were the genes significantly associated with both *in vitro* drug sensitivity and patient survival and preserved consistent expression patterns between the cell lines and EOC patients. These biomarkers, which were discovered by simultaneously utilizing *in vitro* drug sensitivity and patient outcome information, were then used for our prediction modeling of each drug response. Using both principal component and cross-validated regression analyses sequentially on the final biomarker set, we avoided model overfitting with the training NCI-60 set. For practical interpretation and use of our gene expression model prediction values without loss of information, the predicted scores were converted into rank-based percentile scores between zero and unity within each set. Trained models were evaluated with the clinical response and survival data of EOC patients to obtain the best therapeutic predictor for each drug. For this evaluation of competing models, we used the Bonome-185 set for paclitaxel and cyclophosphamide and the TCGA-UW set for topotecan.

The Bonome-185 and the TGGA-UW sets also used to *pre-define* predicted responders (CR) and non-responders (NR) maximizing the Youden’s J index (sensitivity+specificity-1). We conducted a time-dependent receiver operating characteristics (ROC) analysis for an overall survival of 5 years to define optimal cutoff values for high clinical utility by the area under the curve (AUC). The optimal cutoff values for the final predictors were determined by maximizing the Youden’s J index on the ROC curves. These cutoff values were used to stratify patients in the external validation cohorts as if they had been predicted for their therapeutic outcomes prior to treatment. Patients with higher predictor scores than each drug’s cutoff value were considered to be predicted responders to the drug.

For each predictor, an external validation was conducted to confirm its objective predictability for the chemotherapy response and survival of EOC patients. For this external validation, the final predictors of the three drugs were independently applied to EOC cohorts, which were not used for our model development in any manner. Performance of these predictors was first evaluated by testing for a significant difference in the prediction scores between complete response (CR) and other (non-response; NR) patients using a non-parametric Wilcoxon rank-sum test. We then performed a multivariate logistic regression analysis to examine the prediction performance of the clinical response with other clinical variables such as patient age, debulking status, and tumor stage. We also performed Cox proportional hazard regression analyses to understand the prediction performance for patient variable survival times by the three drugs’ predictors together with other important clinical variables.

## Results

### Final Drug Biomarkers and Predictors

The final predictor for paclitaxel was comprised of 20 biomarkers with an AUC of 0.766 for 107 patients treated with the drug in the Bonome-185 cohort (P<0.01). The predictor for cyclophosphamide consisted of 44 genes with an AUC of 0.664 for 68 cyclophosphamide-treated patients also in the Bonome-185 cohort (P = 0.024). As for topotecan, the final predictor included 58 genes with an AUC of 0.917 for 10 patients treated with topotecan in the TCGA-UW cohort (P = 0.143); the Topotecan predictor was not statistically significant due to the small sample size of this cohort despite a very high AUC value (see **Results S1** and **Figure S1** for the detailed gene lists and the ROC analyses).

### Predictor Evaluation with Independent EOC Cohorts

We examined the prediction performance of the above predictors on independent patient sets that were not used for our biomarker discovery and model training. We first examined the stratification performance of paclitaxel predictor scores between patients with CR and NR for two independent cohorts, TCGA-448 and UVA-51, for short-term clinical response to the primary chemotherapy with paclitaxel; note that clinical response information was available only for paclitaxel, since it was used in the primary platinum-based combination chemotherapy for most EOC patients. In our univariate logistic regression analysis for each of the predictors and clinical variables, a highly significant difference was found between the two patient groups in TCGA-448 (p-value = 0.003). For the UVA-51 cohort, paclitaxel predictor scores showed a marginally significant difference between 28 CR and 23 NR patients due to its relatively small sample size (p-value = 0.075, left column in **Table 2**). As widely recognized, we also found that optimal vs. suboptimal debulking status was significantly associated with therapeutic response to the primary chemotherapy treatments. Adjusting for the effects of surgical outcome, age, and tumor stage, multivariate logistic regression analysis also showed that patients with higher predictor scores and optimal debulking had significantly higher chances of therapeutic response (predictor odds ratio [OR] = 3.591; 95% CI: 1.494–8.85; P = 0.005, right column in **Table 2**). Therefore, the predictor showed predictive information beyond patient debulking status in this multivariate analysis. For the UVA-51 cohort, the paclitaxel predictor again showed a marginally significant association with drug response (predictor OR = 9.521; 95% CI: 0.99–125.73, P = 0.063).

**Table 2:** Logistic regression analysis for the paclitaxel prediction of primary chemotherapy response.

		Univariate^a^		Multivariate^b^	
Validation cohort	Variables	Odds ratio (95% CI)	P-value	Odds ratio (95% CI)	P-value
**TCGA-448(n = 351)**	**Predictor Score**	**3.574 (1.567, 8.328)**	0.003***	**3.591 (1.494, 8.85)**	0.005***
	Surgical outcomes(sub vs optimal)	0.313 (0.184,0.531)	<0.001***	0.327 (0.187,0.568)	<0.001***
	Stage (IV vs II–III)	0.85 (0.46, 1.622)	0.611	0.812 (0.413, 1.639)	0.551
	Age	1.002 (0.982,1.024)	0.823	1.003 (0.979, 1.027)	0.796
**UVA-51(n = 51)**	**Predictor Score**	**6.328 (0.884,54.155)**	0.075*	**9.521 (0.1, 125.726)**	0.063*
	Surgical outcomes(sub vs optimal)	0.202 (0.053,0.677)	0.013**	0.183 (0.04, 0.71)	0.019**
	Stage (IV vs III)	0.513 (0.629, 3.375)	0.487	2.303(0.222,24.469)	0.464
	Age	0.957 (0.901, 1.013)	0.14	0.948 (0.88, 1.013)	0.13

a An univariate logistic regression analysis was performed for each of the predictor and clinical variables to predict patient clinical response to paclitaxel; statistical significance was reported with overall model significance p-value.

b A multivariate logistic regression analysis was performed with predictor and all clinical variables in the same model; the statistical significance of each variable was derived from the fitted model.

We next examined the prediction performance of the three drug predictors and clinical variables for long-term survival of the independent EOC patient sets. Both univariate and multivariate Cox regression survival analyses showed that paclitaxel predictor scores were significantly associated with overall survival (OS) and progression-free survival (PFS) times for EOC patients in the TCGA-448 cohort (**Table 3**). Notably, no clinical variables (including debulking status) were significantly associated with long-term survival. We were not able to obtain reliable statistical results in this Cox regression survival analysis for the UVA-51 cohort due to its relatively small sample size. Cyclophosphamide and topotecan are largely used for treating recurrent and progressive EOC patients, so only patient OS information after treatment was available for these drugs. We therefore performed both univariate and multivariate Cox regression analyses using the backward variable elimination process to examine whether the two drugs’ predictor scores and other clinical variables were predictive of OS times. Cyclophosphamide predictor scores were found to be significantly associated with overall survival (HR = 0.127; 95% CI: 0.021–0.745, p = 0.022), while clinical variables such as surgical outcome, tumor stage, and age were not (**Table 3**). Topotecan predictor scores were also significantly associated with overall survival (HR = 0.345; 95% CI: 0.122–0.972, p = 0.044), but the clinical variables were not (**Table 3**).

**Table 3:** Cox regression survival analysis for the prediction of patient survival after primary and secondary chemotherapies.

				Univariate^a^		Multivariate^b^	
Predictor	Cohort	Survivaltime	Variables	Hazard ratio(95% CI)	P-value	Hazard ratio(95% CI)	P-value
**Paclitaxel**	**TCGA-448** **(n = 351)**	PFS	**predictor score**	**0.515(0.332, 0.798)**	0.003**	**0.511(0.323, 0.809)**	0.004***
			Surgical outcome(Sub vs Optimal)	1.099(0.821,1.472)	0.525	1.026(0.757,1.391)	0.868
			Stage(IV vs II–III)	1.14(0.804, 1.615)	0.463	1.121(0.773,1.624)	0.547
			Age	0.998(0.987,1.009)	0.728	0.998(0.987, 1.011)	0.821
		OS	**predictor score**	**0.555(0.347,0.889)**	0.014**	**0.585(0.36, 0.951)**	0.031**
			Surgical outcome(Sub vs Optimal)	1.248(0.922,1.689)	0.152	1.13(0.825, 1.548)	0.446
			Stage (IV vs II–III)	1.051(0.731,1.51)	0.79	1.051(0.715, 1.546)	0.801
			Age	1.014(1.001,1.027)	0.033**	1.012(0.999,1.025)	0.082*
**Cyclophosphamide**	**TCGA-448** **(n = 27)**	OS	**predictor score**	**0.124(0.022,0.702)**	0.018**	**0.127(0.021, 0.745)**	0.022**
			Surgical outcome(Sub vs Optimal)	0.529(0.153, 1.83)	0.314	0.495(0.121, 2.031)	0.329
			Stage (IV vs II–III)	0.359(0.045,2.857)	0.333	–	–
			Age	0.1(0.959, 1.043)	0.986	1.024(0.969, 1.082)	0.404
**Topotecan**	**TCGA-test** **(n = 53)**	OS	**predictor score**	**0.403(0.144,1.124)**	0.083*	**0.345(0.122, 0.972)**	0.044**
			Surgical outcome(Sub vs Optimal)	0.696(0.345,1.401)	0.309	–	–
			Stage (IV vs II–III)	1.132(0.564,2.271)	0.727	1.333(0.655, 2.713)	0.428
			Age	0.023(0.992,1.055)	0.141	1.026(0.994, 1.059)	0.11

a Univariate logistic regression analysis was performed for each of the predictor and clinical variables to predict patient survival after primary and secondary chemotherapies; statistical significance was reported with overall model significance p-value.

b A multivariate Cox regression analysis was performed with the predictor and all clinical variables in the same model; the statistical significance of each variable was derived from the fitted model. Both OS and PFS were predicted after the primary platinum-based chemotherapy with paclitaxel, and OS was predicted after the secondary chemotherapy, either with cyclophosphamide or topotecan.

### Survival Difference between Predicted Responders and Non-responders among Recurrent EOC Patients

**Figure 2:**
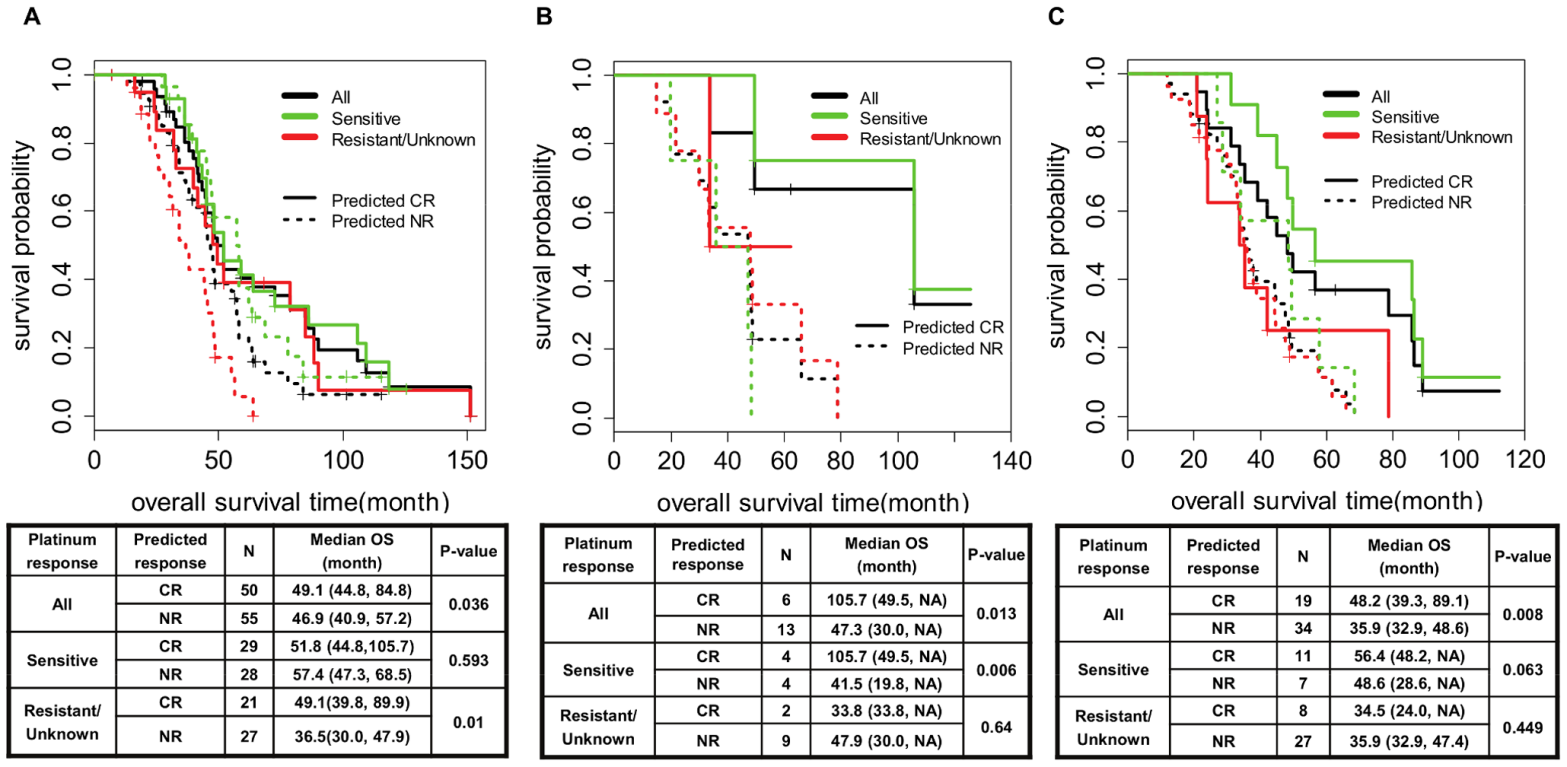
Kaplan-Meier survival analysis of predicted responders and nonresponders among recurrent EOC patients. (A) paclitaxel predictor prediction for OS in TCGA-448, (B) cyclophosphamide predictor prediction for OS in TCGA-448, (C) topotecan predictor prediction for OS in TCGA-test.

We next evaluated the survival time difference between predicted responders (CRs) and non-responders (NRs) among patients treated with one of the three drugs after their disease recurrence by Kaplan-Meier (KM) survival and ROC analyses. In particular, this survival analysis was evaluated for all recurrent patients as well as separately for platinum-sensitive and platinum-resistant patients (defined from the primary chemotherapy response) as these two subgroups of patients show quite different disease outcomes and survival. The predefined cutoff value of each drug predictor was used to score each drug’s responders and non-responders. A patient with a higher predictor score than the cutoff value of the drug was considered to be a predicted responder to the drug. KM survival distributions of these two groups are shown for platinum-sensitive and platinum-resistant patients in **Figure 2**. For the paclitaxel predictor prediction for 105 patients treated with this drug after recurrence, the median overall survival time was 49.1 months (95% CI: 44.8–84.8) among the 50 predicted CR patients compared with 46.9 months (95% CI: 40.9–57.2) among the 55 predicted NR patients (log-rank test p-value = 0.036) (**Figure 2 A** **; Figure S2** for all, platinum-sensitive, and –resistant groups separately). The median survival times were not much different with 51.8 months vs. 57.4 months for the predicted CR and NR patients within the platinum-sensitive patient subgroup, but somewhat surprisingly 39.8 months vs. 36.5 months for the predicted CR and NR groups within the platinum-resistant/unknown patient subgroup. The median PFS time was 18.9 months (95% CI: 17.6–21.2) of the predicted CR patients was also significantly longer than 15.3 months (95% CI: 13.9–17.6) of the predicted NR patients (log-rank test p-value = 0.004). As for the UVA-51 cohort, the median overall survival time was 90.2 months (95% CI: 33.6–NA) for the 21 predicted responders and 37.2 months (95% CI: 22.7–72.6) for the 30 predicted non-responders (log-rank test p-value = 0.163, **Figure S3 A**), and the median progression-free survival time of the predicted responders was 16.3 months (95% CI: 11.84–83.3) and 10.6 months (95% CI: 8.55–14.6) for the predicted non-responders (log rank test p-value = 0.048, **Figure S3 B**); we did not perform the platinum subgroup analysis for this cohort due to its small sample size. Thus, similar survival benefits were observed for both cohorts, even though the statistical significance is weaker for the latter cohort due to its relatively small sample size.

As for cyclophosphamide, the predefined cutoff value of 0.647 provided an AUC of 0.733. This cutoff separated 19 patients who received cyclophosphamide as their second-line treatment into 6 predicted CRs and 13 predicted NRs in the TCGA set. The median overall survival time of the predicted CR patients was 105.7 months and that of the predicted NR patients was 47.3 months, which was statistically significant despite the small sample size (log-rank test p-value = 0.013, **Figure 2 B** **and Figure S4**); the median OS times were 105.7 months vs. 41.5 months for the predicted CR and NR patients within the platinum-sensitive patient subgroup and 33.8 months vs. 47.9 months within the platinum-resistant subgroup. Finally, for topotecan, the cutoff value of 0.766 resulted in an AUC of 0.91 for 19 predicted CR and 34 predicted NR patients from the TCGA-test data. The median overall survival time of the predicted CRs was 48.2 months and that of the predicted NRs was 35.9 months (log-rank test p-value = 0.008, **Figure 2 C** **and Figure S5**); the median OS times were 56.4 months vs. 48.6 months for the predicted CR and NR patients within the platinum-sensitive patient subgroup and 34.5 months vs. 35.9 months within the platinum-resistant subgroup. Finally, in order to assure our predictors were not merely prognostic predictors, we examined whether our predictor stratification resulted in improved survival for patients who were not treated with each of the three drugs and confirmed that there was no survival difference between predicted CR and NR patients if they were not treated with the specific drug of prediction (**Figure S6**).

### Expected Clinical Benefit from Biomarker-guided Chemotherapy

While the previous analyses showed the predictive power of our predictors for both patient therapeutic response and survival, it is of great interest to assess the expected clinical benefit when the three drugs’ predictors are utilized together for individual patients with personalized treatment selection. We can objectively assess such an expected clinical benefit in a historical patient cohort as follows. First, in order to make a potential drug selection strategy for individual patients based on the predictor scores of the three standard chemotherapy drugs, the “*comparative effectiveness*” of these drugs relative to their predictor prediction scores needed to be understood. Therefore, using the large TCGA-448 cohort from >10 diverse clinical centers, we estimated positive predictive values (PPVs) for the probability of five-year survival across varying cutoff values of the three drug predictor scores (**Figure S7**). These PPVs provided us with the comparative statistical chances of five-year survival from the therapeutic response predictions by the three drug predictors. As shown in **Figure S7**, the PPVs rose significantly, from 20% to near 50%, as each drug’s predictor values were increased. Then, using the three drugs’ predicted predictor scores for individual recurrent EOC patients, we determined which drug would have been most beneficial for each patient of the TCGA-448 cohort, that is, which drug provided the highest statistical chance of five-year survival based on the patient’s predictor scores for the three drugs.

Based on this drug selection strategy, we found that 308 EOC patients in the TCGA-448 data set were, in fact, treated in their primary chemotherapy with one of the three drugs (most with paclitaxel). Among them 93 patients were found to be treated with COXEN matched drugs with the highest PPVs based on our predictions, whereas 215 patients were not; we refer to the former group as COXEN biomarker “matched” and the latter as COXEN biomarker “unmatched.” We carefully examined whether there were any differences in any important clinical characteristics such as tumor stage, age and predictor score distributions between the matched and unmatched groups. We found these properties were almost identical with no statistical difference in known prognostic factors such as tumor stage and others (data not shown). Therefore, we could safely consider that the patient prognostic factors independent of the treatments were equivalent between the two groups and that the differences between the two groups’ therapeutic and survival outcomes could be explained mainly by their treatment selections.

Note that almost all patients were treated with paclitaxel in their primary platinum-based chemotherapy, so the matched patients were largely those who were predicted to have the highest benefit from this taxane agent and the unmatched patients were those who were predicted to have a lower benefit from this drug than the other drugs. We found that the drug response rate of the COXEN-matched group was 79.3%, which was significantly higher than the 66.9% of the unmatched group (binomial test p-value = 0.05, **Table 4**). Therefore, the two groups of patients were treated with the same first-line chemotherapy, but the response rate among the matched patients was significantly higher than that of the unmatched patients, even in their primary chemotherapy.

**Table 4:** Clinical response rates of COXEN-matched vs. unmatched patient groups in the TCGA cohort after the primary platinum-based chemotherapy.

		Drug response after first-line chemotherapy			
Drug Assignment	COXEN Guided drug	Responder (row %)	Nonresponder	Missing	Total
Matched	Paclitaxel^a^	64(80.0%)	16	10	90
	Cyclophosphamide	1(50%)	1	1	3
	Topotecan	–	–	–	–
	Subtotal	65(79.3%)	17	111	93
Unmatched	Paclitaxel	0	1	–	1
	Cyclophosphamide	111 (67.7%)	53	31	195
	Topotecan	10 (62.5%)	6	3	19
	Subtotal	121 (66.9%)	60	34	215

a Almost all patients were treated with paclitaxel in the first-line chemotherapy, so the matched patients were predicted to have the highest survival benefit from the drug (of the three) and the unmatched patients were predicted to have the highest survival benefit from the other two drugs.

**Figure 3:**
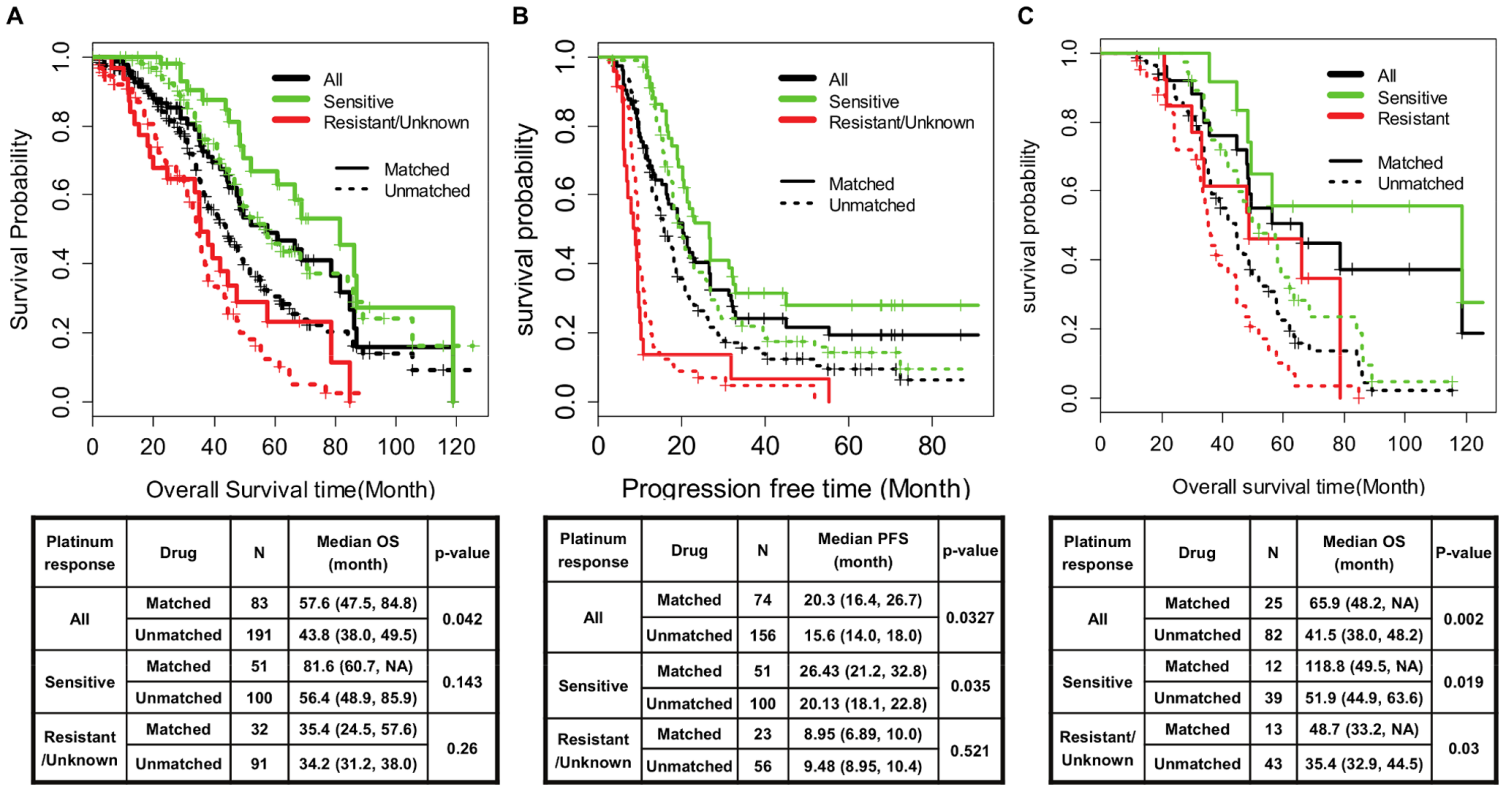
Kaplan-Meier survival stratification between COXEN-matched and unmatched patients in the TCGA-448 cohort. (A) OS difference between matched and unmatched patients, (B) PFS difference between matched and unmatched patients, (C) OS difference between matched and unmatched patients among recurrent EOC patients.

We then compared overall survival (OS) and progression-free survival (PFS) benefits between the COXEN-matched and unmatched groups among 274 patients treated with one of the three drugs in their primary and subsequent chemotherapies with follow-up survival information (**Figure 3**). These survival benefits were evaluated for all 274 patients as well as separately for platinum-sensitive and platinum-resistant patients as the two subgroups show quite different survival outcomes (excluding 34 patients too early to define their platinum response). The median overall survival time of the COXEN-matched group was 57.6 months, which was significantly longer than the 43.8 months for the unmatched group (log-rank test p-value = 0.042, **Figure 3 A** **and Figure S8**); the median OS times were 81.6 months vs. 56.4 months for the matched and unmatched patients within the platinum-sensitive patient subgroup and 35.4 months vs. 34.2 months within the platinum-resistant subgroup. Similarly, the median PFS time of the matched group was 20.3 months compared with 15.6 months for the unmatched group (log-rank test P-value = 0.033, **Figure 3 B** **and Figure S9**); the median PFS times were 26.4 months vs. 20.1 months for the matched and unmatched patients within the platinum-sensitive patient subgroup and 8.9 months vs. 9.4 months within the platinum-resistant subgroup. We also examined the survival outcomes for the patients treated with one of the three drugs after their recurrent disease. Of 107 recurrent patients treated with one of the three drugs, 25 patients were treated with COXEN-matched drugs and 82 patients with other drugs. Median overall survival times were 65.9 and 44.2 months for the matched and unmatched groups, respectively (log-rank test p-value = 0.002, **Figure 3 C** **and Figure S10**); the median OS times were 118.8 months vs. 51.9 months of the matched and unmatched patients within the platinum-sensitive patient subgroup and 48.7 months vs. 35.4 months within the platinum-resistant subgroup. Therefore, the recurrent EOC patients in the matched group survived 21 months longer than the patients in the unmatched group. Also, the platinum-response subgroup analyses showed that survival improvement was greater for platinum-sensitive patients if they were treated with the predicted most beneficial drugs than platinum-resistant patients.

## Discussion

Despite the availability of multiple standard chemotherapy drugs and the recent advent of targeted therapeutic agents, the overall therapeutic response and survival of advanced EOC patients has not improved much over the last two decades. Advanced EOC patients are highly heterogeneous in their therapeutic responses, so only a small fraction of the patient population responds to each standard therapeutic option. Consequently, if existing and novel drugs are unselectively administered to individual patients, overall therapeutic outcome of advanced EOC is difficult to improve. In this study, we have obtained the single-drug COXEN predictors by integrating each drug’s *in vitro* drug activity data and patient therapeutic outcome information for three standard chemotherapy drugs used in treating advanced EOC: paclitaxel, cyclophosphamide, and topotecan, for which multiple independent patient data sets were available for our stringent statistical modeling, evaluation, and external testing. In particular, our initial biomarker discovery step from *in vitro* single-drug sensitivity data enabled us to identify gene expression biomarkers which were associated with each single drug sensitivity and independent of other known biological factors correlated with drug response. A further triage of these initial biomarkers for direct association with patient survival then allowed us to select the final single-drug biomarkers that were also relevant to human patient outcomes.

We first found that these COXEN predictors independently showed high prediction for both patient short-term therapeutic response and long-term survival. We then examined the potential benefit from a personalized treatment use of the three drug predictors on the TCGA cohort of 443 EOC patients from >10 clinical centers. Following the FDA guideline for statistical evaluation of diagnostic predictors (FDA Docket No. 2003D-0044), we independently examined these predictors in a prospective manner on the patient tumors from the primary surgery prior to systemic therapy. From this prospective testing we found that both overall survival and PFS of the cohort were significantly prolonged when patients had been treated with the predicted most beneficial drugs. When this benefit was examined for patients with recurrent EOC, overall survival was 21 months longer for the patients treated with the drugs predicted to be the most beneficial drugs (COXEN-matched) than the patients treated with other drugs (COXEN-unmatched). Survival improvement was greater for the platinum-sensitive patients than the platinum-resistant patients.

We closely examined whether our COXEN models were merely prognostic predictors that selected patients simply with longer survival irrelevant to the specific drug treatments. We directly investigated this issue by comparing the survival difference among the patients who were not treated with the respective drugs, and confirmed that even if patients had higher COXEN scores, they showed neither better therapeutic response nor longer survival unless they were treated with the predicted effective drugs. Also, in order to examine whether these survival difference observed by the COXEN stratification was due to any other confounding factors, we compared the distributions of all available clinicopathological variables such as tumor stage and age between the COXEN matched and unmatched groups, and found that the two subgroups had identical distributions for all these variables. Therefore, the COXEN matched group showed a significantly longer survival than the unmatched group while both groups had identical clinical characteristics except that the former group was treated with the predicted effective drugs. Avoiding potential bias on a cohort from a single site, we thus believe that our observations on the TCGA cohort from >10 clinical centers could reasonably reflect the outcomes from the use of these predictors on the general EOC patient population. Also, note that the patient characteristics and survival statistics of this TCGA cohort have been confirmed to be well matched with those in the general EOC population [14].

It is worthwhile to note several limitations of our current study. In this study we were able to perform our COXEN analysis only on the three standard chemotherapy drugs for which we had multiple patient data sets for our rigorous statistical prediction modeling, independent evaluation, and external validation. We employed this strict statistical principle to avoid many pitfalls from a genomic-based biomarker study, which resulted in a very limited set of drugs for our analysis. Despite such a limitation, we found that a comparative effectiveness-based selection only among the three drugs could still potentially provide a survival benefit compared to the current unselective use of many standard agents for recurrent EOC. Thus, we believe that, if further validated in a prospective setting, this kind of comparative drug selection strategy based on multiple therapeutic biomarker predictors may be proven to be highly effective to improve patient outcomes. This can then be expanded to a more comprehensive prediction capability among other commonly used chemotherapy agents, such as liposomal doxorubicin, and even different administration schedules, including weekly paclitaxel. Unfortunately, current patient data with which we can assess such comparative effectiveness are very limited. As such, our study was based only on the estimated efficacy among limited drug selections. Also, our statistical estimation of the positive predictive value (PPV) curves for the drug predictors could be further improved by a non-parametric estimation to correlate their predicted scores more precisely with patient 5-year survival probabilities if larger numbers of patients were available for these drugs. Finally, we note that even if our retrospective analysis has showed some evidence for an improved survival of advanced EOC by a selective use of several standard chemotherapy drugs, these findings must be confirmed in a prospective study, which may also allow us to refine our comparative drug selection strategy among the drugs.

## Supporting Information

Figure S1 **ROC and AUC analysis of 3 final predictors (A) ROC analysis of paclitaxel prediction of 107 patients in Bonome cohort, (B) ROC of cyclophosphamide prediction of 68 patients in Bonome cohort, (C) ROC of topotecan prediction of 41 patients in TCGA-UW.**
(TIF)

Figure S2 **Kaplan-Meier survival analysis of predicted responders and nonresponders among recurrent EOC patients treated with paclitaxel.** (A) all patients, (B) platinum-sensitive patients, (C) platinum-resistant patients.
(TIF)

Figure S3 **Kaplan-Meier survival analysis of predicted responders and nonresponders in independent patient cohorts.** (A) paclitaxel predictor prediction for OS in UVA-51, (B) paclitaxel predictor prediction for PFS in UVA-51.
(TIF)

Figure S4 **Kaplan-Meier survival analysis of predicted responders and nonresponders among recurrent EOC patients treated with cyclophosphamide.** (A) all patients, (B) platinum-sensitive patients, (C) platinum-resistant patients.
(TIF)

Figure S5 **Kaplan-Meier survival analysis of predicted responders and nonresponders among recurrent EOC patients treated with topotecan (A) all patients, (B) platinum-sensitive patients, (C) platinum-resistant patients.**
(TIF)

Figure S6 **Kaplan-Meier survival analysis for the validation of not being prognostic prediction on patients not treated with each drug.** (A) paclitaxel predictor prediction, (B) cyclophosphamide predictor prediction, (C) topotecan predictor prediction.
(TIF)

Figure S7 **Comparative effectiveness of the COXEN predictors.** Five-year survival positive predicted values (PPVs) are plotted against the predictor cutoff values. Paclitaxel and cyclophosphamide predictors provided higher five-year survival chances (PPVs) than topotecan predictors when a patient had similar scores for the three predictors.
(TIF)

Figure S8 **Kaplan-Meier overall survival stratification between COXEN-matched and unmatched patients in the TCGA-448 cohort.** (A) all patients (B) platinum-sensitive patients, (C) platinum-resistant patients.
(TIF)

Figure S9 **Kaplan-Meier progression-free survival stratification between COXEN-matched and unmatched patients in the TCGA-448 cohort.** (A) all patients (B) platinum-sensitive patients, (C) platinum-resistant patients.
(TIF)

Figure S10 **Kaplan-Meier overall survival stratification between COXEN-matched and unmatched patients in the recurrent EOC patients in TCGA-448 cohort.** (A) all patients (B) platinum-sensitive patients, (C) platinum-resistant patients.
(TIF)

Methods S1 **Supporting methods.**
(DOCX)

Results S1 **Supporting results.**
(ZIP)
