# Supplementary figures and images for: Retrospective Analysis of Survival Improvement by Molecular Biomarker-Based Personalized Chemotherapy for Recurrent Ovarian Cancer
Source: PLoS One. 2014 Feb 5;9(2):e86532. doi: 10.1371/journal.pone.0086532 (PMC3914805; doi:10.1371/journal.pone.0086532)

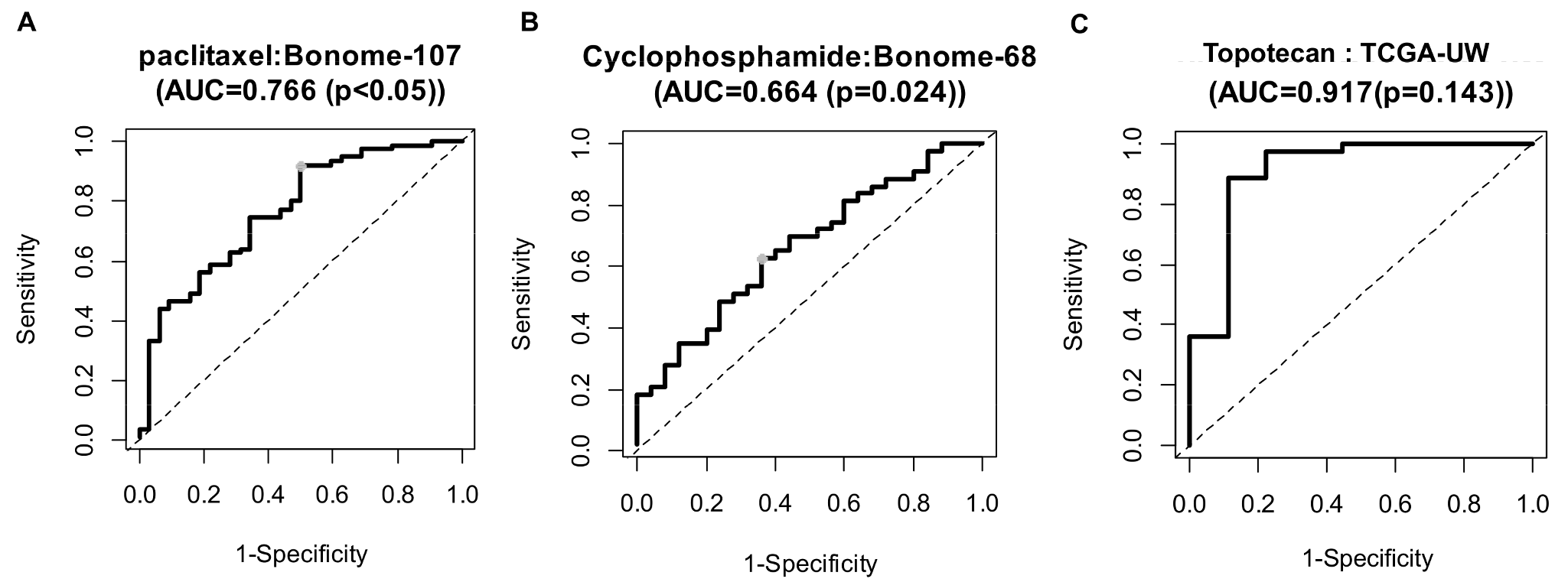

Supplement: Figure S1 — ROC and AUC analysis of 3 final predictors (A) ROC analysis of paclitaxel prediction of 107 patients in Bonome cohort, (B) ROC of cyclophosphamide prediction of 68 patients in Bonome cohort, (C) ROC of topotecan prediction of 41 patients in TCGA-UW. (TIF) [file pone.0086532.s001.tif]

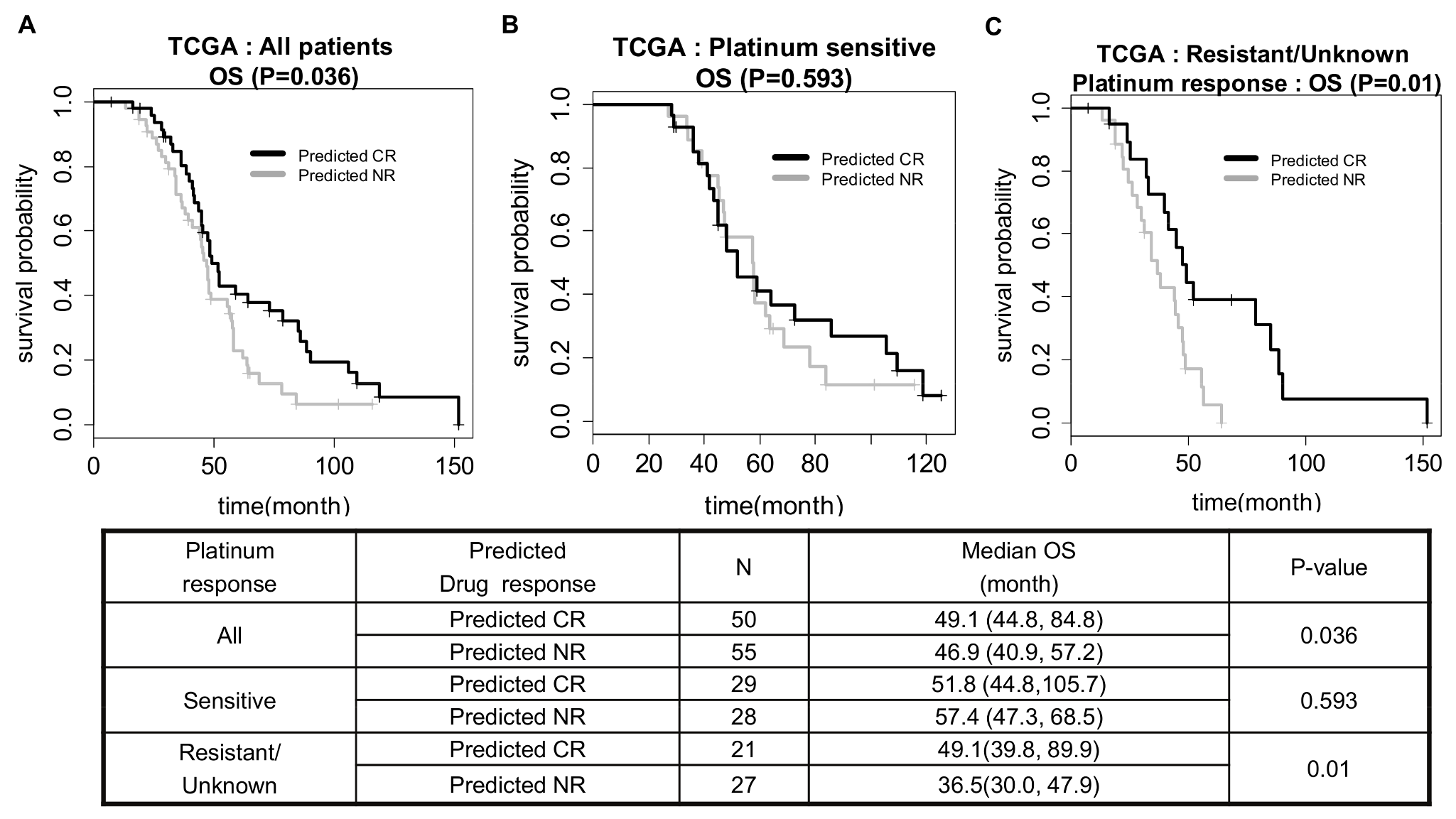

Supplement: Figure S2 — Kaplan-Meier survival analysis of predicted responders and nonresponders among recurrent EOC patients treated with paclitaxel. (A) all patients, (B) platinum-sensitive patients, (C) platinum-resistant patients. (TIF) [file pone.0086532.s002.tif]

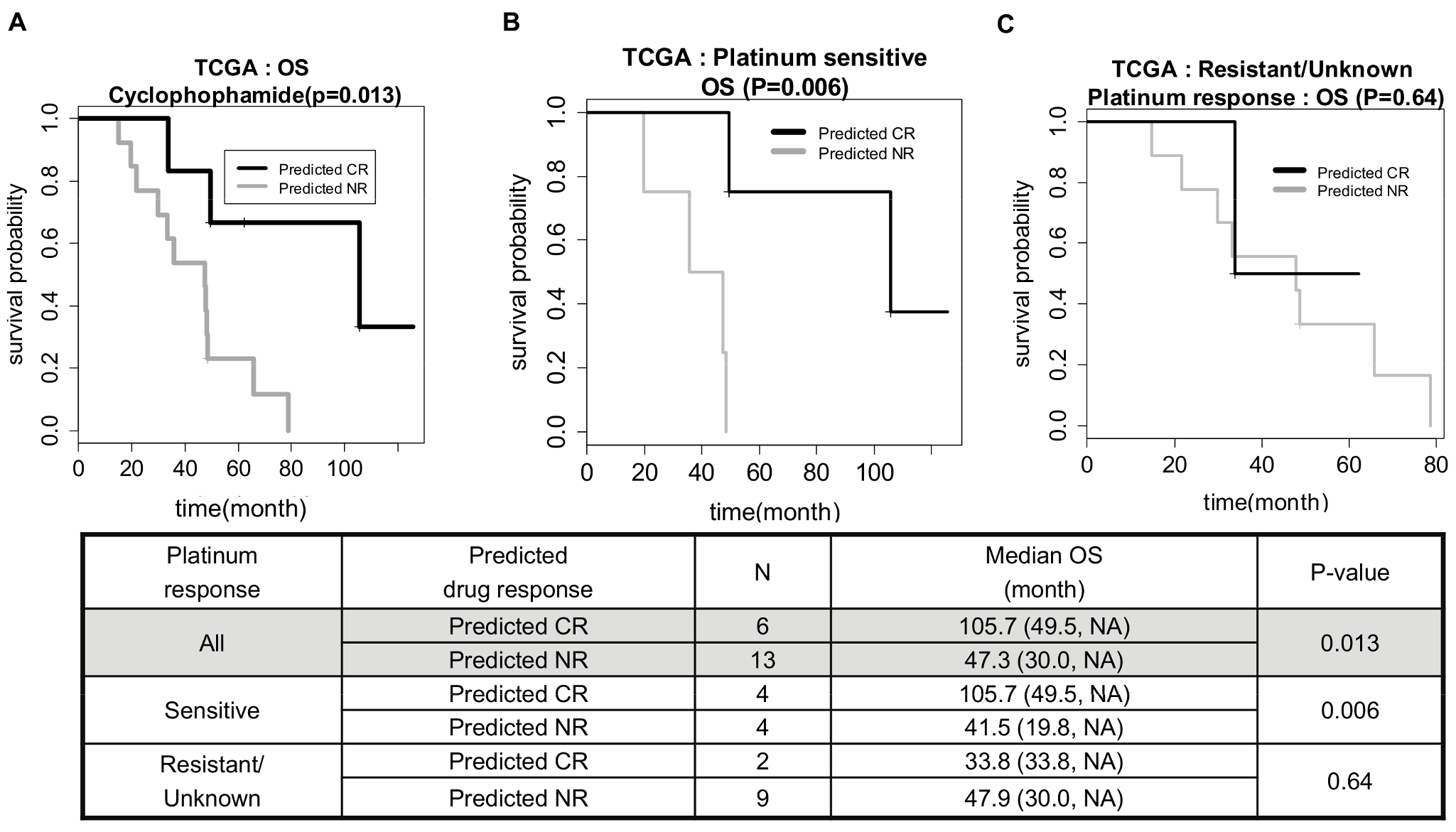

Supplement: Figure S4 — Kaplan-Meier survival analysis of predicted responders and nonresponders among recurrent EOC patients treated with cyclophosphamide. (A) all patients, (B) platinum-sensitive patients, (C) platinum-resistant patients. (TIF) [file pone.0086532.s004.tif]

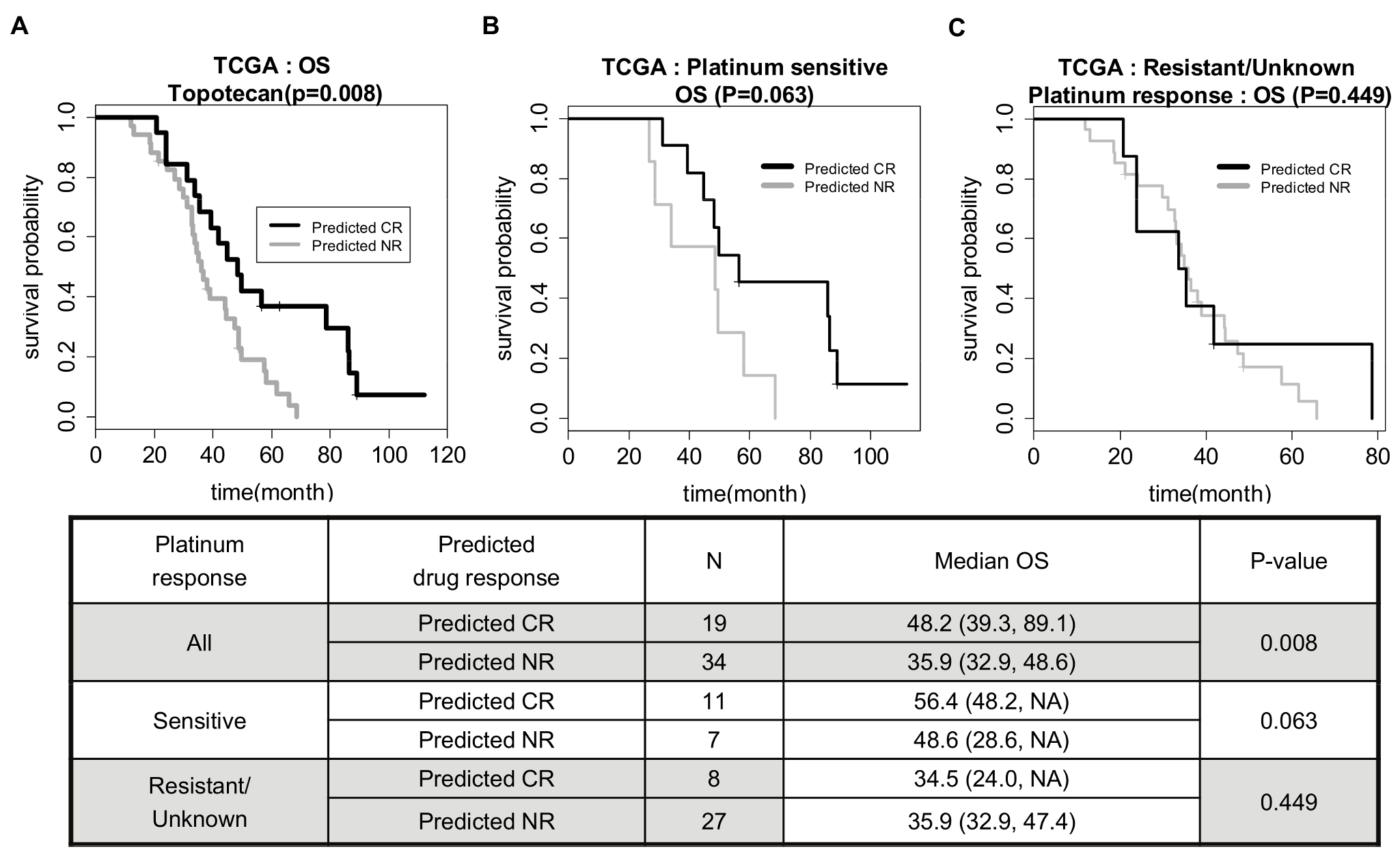

Supplement: Figure S5 — Kaplan-Meier survival analysis of predicted responders and nonresponders among recurrent EOC patients treated with topotecan (A) all patients, (B) platinum-sensitive patients, (C) platinum-resistant patients. (TIF) [file pone.0086532.s005.tif]

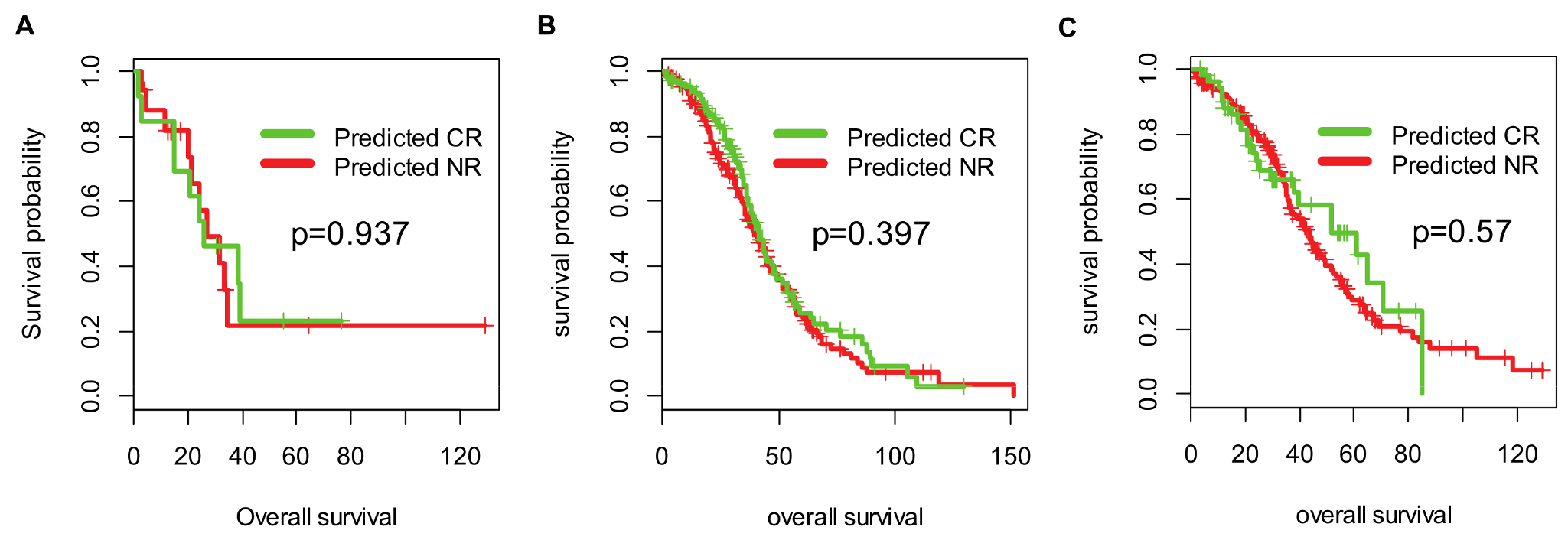

Supplement: Figure S6 — Kaplan-Meier survival analysis for the validation of not being prognostic prediction on patients not treated with each drug. (A) paclitaxel predictor prediction, (B) cyclophosphamide predictor prediction, (C) topotecan predictor prediction. (TIF) [file pone.0086532.s006.tif]

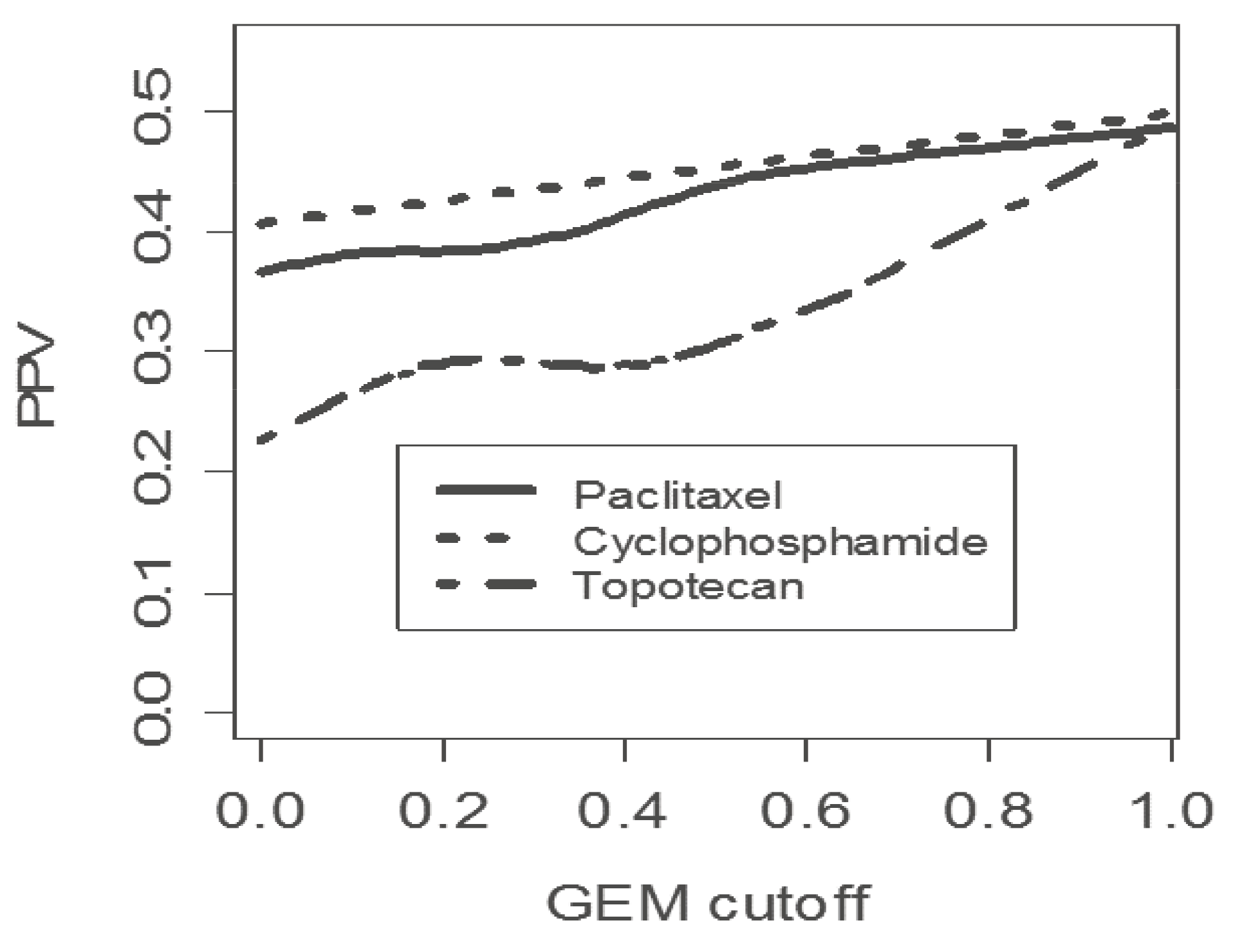

Supplement: Figure S7 — Comparative effectiveness of the COXEN predictors. Five-year survival positive predicted values (PPVs) are plotted against the predictor cutoff values. Paclitaxel and cyclophosphamide predictors provided higher five-year survival chances (PPVs) than topotecan predictors when a patient had similar scores for the three predictors. (TIF) [file pone.0086532.s007.tif]

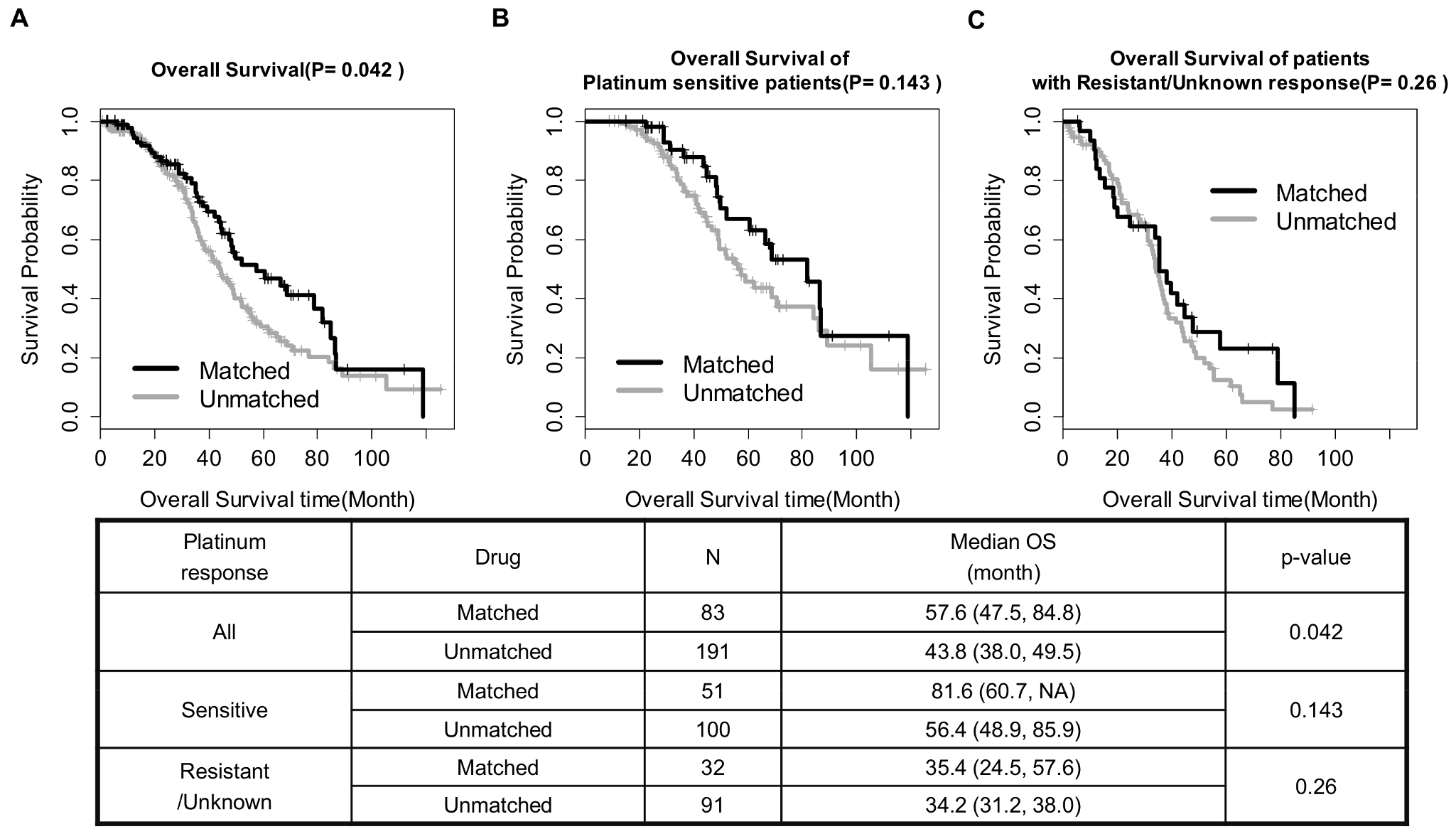

Supplement: Figure S8 — Kaplan-Meier overall survival stratification between COXEN-matched and unmatched patients in the TCGA-448 cohort. (A) all patients (B) platinum-sensitive patients, (C) platinum-resistant patients. (TIF) [file pone.0086532.s008.tif]

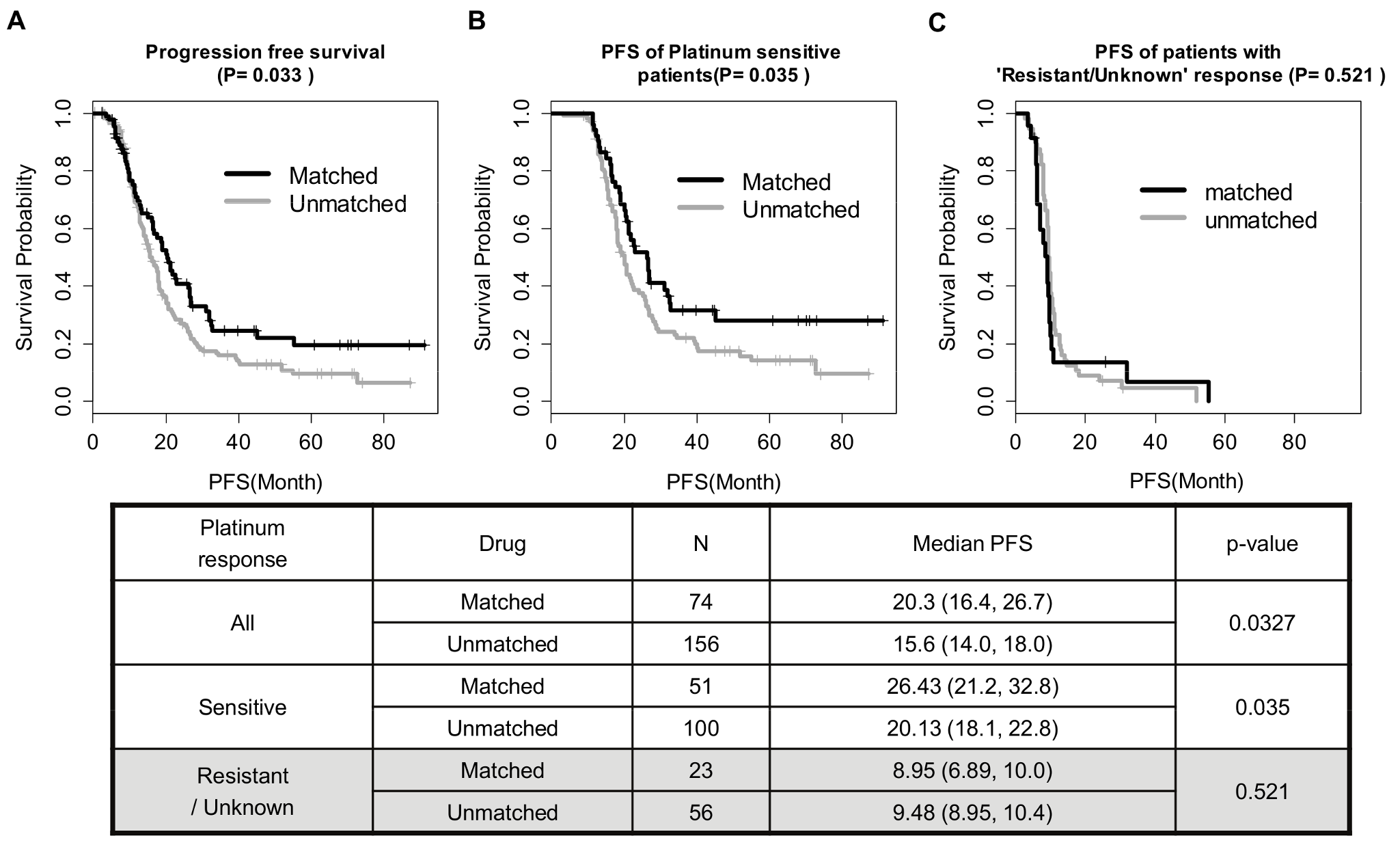

Supplement: Figure S9 — Kaplan-Meier progression-free survival stratification between COXEN-matched and unmatched patients in the TCGA-448 cohort. (A) all patients (B) platinum-sensitive patients, (C) platinum-resistant patients. (TIF) [file pone.0086532.s009.tif]

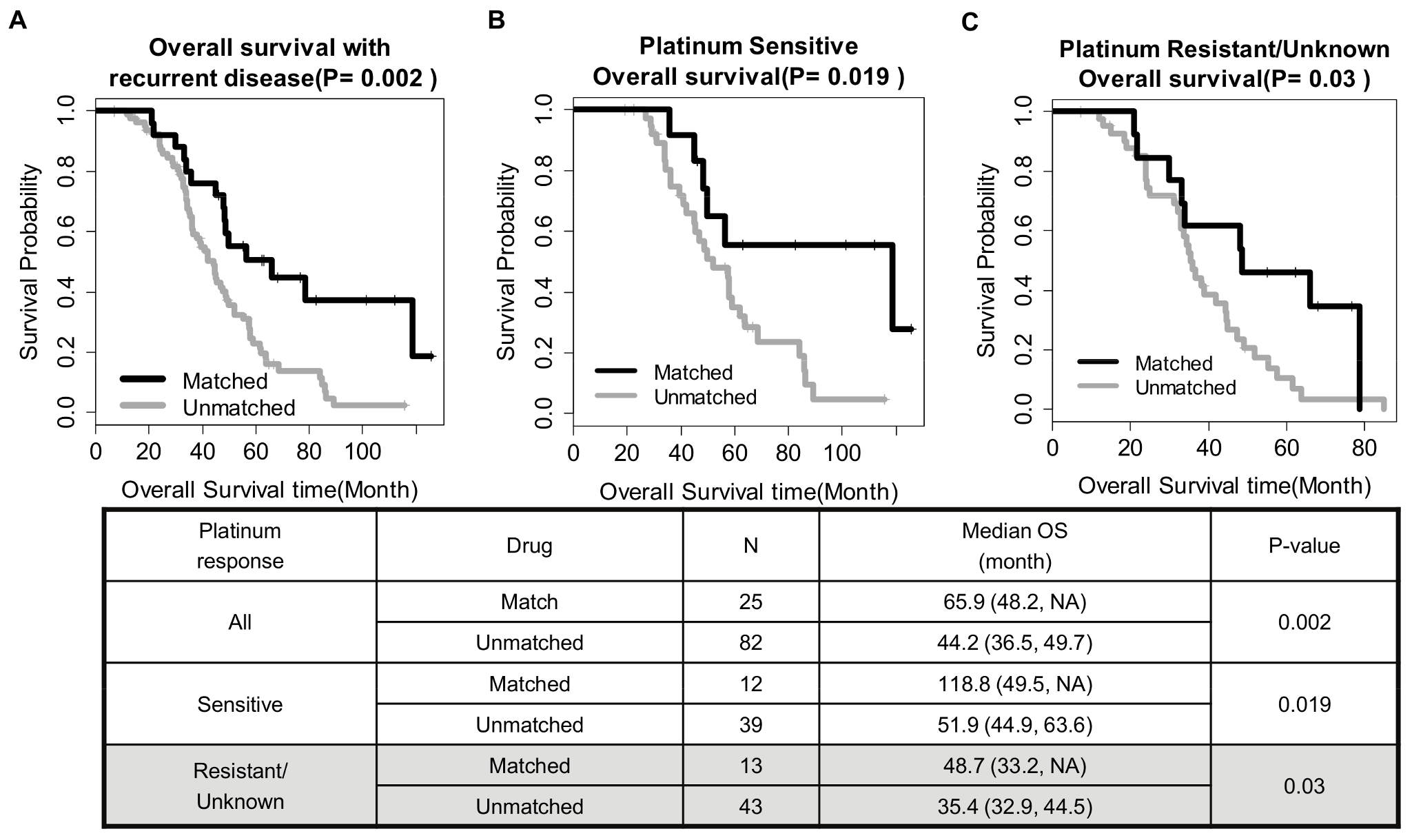

Supplement: Figure S10 — Kaplan-Meier overall survival stratification between COXEN-matched and unmatched patients in the recurrent EOC patients in TCGA-448 cohort. (A) all patients (B) platinum-sensitive patients, (C) platinum-resistant patients. (TIF) [file pone.0086532.s010.tif]
