# Supplementary material for: Retrospective Analysis of Survival Improvement by Molecular Biomarker-Based Personalized Chemotherapy for Recurrent Ovarian Cancer
Source: PLoS One. 2014 Feb 5;9(2):e86532. doi: 10.1371/journal.pone.0086532 (PMC3914805; doi:10.1371/journal.pone.0086532)
Supplement: Methods S1 — Supporting methods. (DOCX) [file pone.0086532.s011.docx]

supplementary methods

**Patient Data:** *In vitro* drug activity and microarray data for the 60 NCI cancer cell lines (NCI-60) were previously described [10]. In brief, publicly available drug sensitivity data for 50% growth inhibition (GI_50_) for the NCI-60 were obtained from the NCI Developmental Therapeutics Program (<http://dtp.nci.nih.gov>). NCI-60 expression profiling data with HG-U133A GeneChip® arrays (Affymetrix, Santa Clara, CA) were also obtained from the National Cancer Institute (http://discover.nci.nih.gov). Microarray gene expression data for frozen tissue samples obtained at the time of primary cytoreductive surgery from two large human ovarian cancer cohorts were used for the development and independent evaluation of our drug-response predictors. Clinical follow-up information after surgery and chemotherapy were fully available for these cohorts. The first cohort of 185 EOC patients treated with adjuvant chemotherapy was originally obtained for identifying prognostic molecular signatures of survival [11]. Of 185 patients, 112 (67%) showed complete response (CR), 41 (25%) partial response (PR), 14 (8%) progression of disease (PD), and 18 had unrecorded responses to the primary chemotherapy (**Table 1**). The second set of 448 epithelial ovarian cancer patients with gene expression profiling data and comprehensive clinical data was obtained from **The Cancer Genome Atlas** (TCGA) consortium (<http://tcga-data.nci.nih.gov>) [12]. These EOC patients from >10 diverse clinical centers had received primary platinum-based chemotherapy after surgery. The primary chemotherapy responses of this cohort were comprised of 272 (60.71%) patients with CR, 54 with PR, 25 with stable disease (SD), and 36 with PD. However, a majority of the patients experienced recurrence or progression of disease and so were subsequently treated with additional chemotherapy drugs such as cyclophosphamide and topotecan. In particular, of 100 recurrent patients treated with topotecan, 47 patients were from the University of Washington (TCGA-UW) and the remaining 53 patients were from 11 other hospitals (TCGA-test). For the third cohort of 51 patients with stage III–IV EOC at the University of Virginia (UVA-51), gene expression data were obtained from archived FFPE tissue blocks, and both chemotherapy response and long-term survival information were available [13]. This cohort had 28 CR and 23 NR patients. The last cohort of 99 patients used in our study was from a gene expression profiling study on a general EOC patient population prior to primary chemotherapy; we used this set to find initial biomarkers that were concordantly expressed between cancer cell lines and human patients [10]. More detailed clinical characteristics of these cohorts are summarized in **Table 1**.

**Statistical Analysis:** Multivariate models for predicting patient therapeutic responses to three chemotherapy drugs, paclitaxel, cyclophosphamide, and topotecan, were derived by integrating *in vitro* drug sensitivity data for the NCI-60 cell lines and clinical outcome information from EOC patients after standard chemotherapy. The schematic procedures for our model training and validation are summarized in **Figure 1**. First, initial gene expression biomarkers highly associated with *in vitro* drug sensitivity were identified from the NCI-60 microarray data by correlating each drug’s GI_50_ values for the NCI-60 with their genomic expression data for cyclophosphamide and topotecan treatment and by identifying differentially expressed biomarkers between sensitive and resistant cell lines of the NCI-60 to paclitaxel. These chemosensitivity biomarkers were then triaged based on the COXEN coefficient, which represents the degree of concordance of expression regulation between the NCI-60 cell lines and a general EOC patient population prior to standard chemotherapy [14]. In brief, derivation of the COXEN coefficient is based on a so-called “correlation of correlations,” which first calculates the expression correlations within each set for the identical set of genes and then evaluates gene-by-gene correlation between the correlation matrices of the two sets. This kind of second-order correlation has proven useful by us and others for investigating various gene networks to identify concordant data sets [15-17]. More detailed description of the COXEN algorithm can be found elsewhere [7, 10].

The above biomarkers were further screened with ovarian cancer patient data: the Bonome-185 set for paclitaxel and cyclophosphamide and the TCGA-UW set for topotecan. A subset of each drug’s biomarkers significantly associated with patient survival was identified by a Cox regression survival analysis. Therefore, these final biomarkers were the genes significantly associated with both *in vitro* drug sensitivity and patient survival and preserved consistent expression patterns between the cell lines and EOC patients. These biomarkers, which were discovered by simultaneously utilizing *in vitro* drug sensitivity and patient outcome information, were then used for our prediction modeling of each drug response. Using both principal component and cross-validated regression analyses sequentially on the final biomarker set, we avoided model overfitting with the training NCI-60 set. For practical interpretation and use of our gene expression model (GEM) prediction values without loss of information, the predicted scores were converted into rank-based percentile scores between zero and unity within each set. Trained models were evaluated with the clinical response and survival data of EOC patients to obtain the best therapeutic prediction GEM for each drug. For this evaluation of competing models, we used the Bonome-185 set for paclitaxel and cyclophosphamide and the TCGA-UW set for topotecan.

The Bonome-185 and the TGGA-UW sets also used to *pre-define* predicted responders (CR) and non-responders (NR) maximizing the Youden’s J index (sensitivity+specificity-1). We conducted a time-dependent receiver operating characteristics (ROC) analysis for an overall survival of 5 years to define optimal cutoff values for high clinical utility by the area under the curve (AUC). The optimal cutoff values for the final GEMs were determined by maximizing the Youden’s index on the ROC curves. These cutoff values were used to stratify patients in the external validation cohorts as if they had been predicted for their therapeutic outcomes prior to treatment. Patients with higher GEM scores than each drug’s cutoff value were considered to be predicted responders to the drug.

For each GEM predictor, an external validation was conducted to confirm its objective predictability for the chemotherapy response and survival of EOC patients. For this external validation, the final GEMs of the three drugs were independently applied to EOC cohorts, which were not used for our model development in any manner. Performance of these GEMs was first evaluated by testing for a significant difference in the GEM scores between complete response (CR) and other (non-response; NR) patients using a non-parametric Wilcoxon rank-sum test. We then performed a multivariate logistic regression analysis to examine the GEM prediction performance of the clinical response with other clinical variables such as patient age, debulking status, and tumor stage. We also performed Cox proportional hazard regression analyses to understand the prediction performance for patient variable survival times by the three drugs’ GEMs together with other important clinical variables.
